# Adverse Outcomes of Perioperative Red Blood Cell Transfusions in Coronary Artery Bypass Grafting in Hospital Universiti Sains Malaysia

**DOI:** 10.21315/mjms2019.26.3.4

**Published:** 2019-06-28

**Authors:** Choon Hua Chan, Ghazali Mohamad Ziyadi, Mamat Ahmad Zuhdi

**Affiliations:** 1 Department of Surgery, School of Medical Sciences, Universiti Sains Malaysia, Kubang Kerian, Kelantan, Malaysia; 2 Unit of Cardiothoracic Surgery, Department of Surgery, School of Medical Sciences, Universiti Sains Malaysia, Kubang Kerian, Kelantan, Malaysia

**Keywords:** adverse outcomes, morbidity, red blood cell, perioperative transfusions, CABG, Malaysia

## Abstract

**Background:**

Perioperative red blood cell (RBC) transfusion in coronary artery bypass grafting (CABG) has both benefits and harms. Our aim was to study the association between perioperative RBC transfusion and its adverse outcomes.

**Methods:**

This was a retrospective study of patients who underwent isolated CABG in Hospital Universiti Sains Malaysia, Kelantan, Malaysia, from 1 January 2013 until 31 December 2017. Data were collected from medical records, and comparisons were made between patients who received perioperative RBC transfusions and those who did not have adverse outcomes after CABG.

**Results:**

A total of 108 patients who underwent isolated CABG were included in our study, and 78 patients received perioperative RBC transfusions. Patients who received perioperative RBC transfusions compared to those who did not were significantly more likely to develop prolonged ventilatory support (21.8% versus 0%, *P* = 0.003), cardiac morbidity (14.1% versus 0%, *P* = 0.032), renal morbidity (28.2% versus 3.3%, *P* = 0.005) and serious infection (20.5% versus 3.3%, *P* = 0.037). With each unit of packed RBC transfusions, there was a significantly increased risk of prolonged ventilatory support (adjusted odds ratio [AOR] = 1.45; 95% confidence interval [CI] = 1.20–1.77; *P <* 0.001), cardiac morbidity (AOR =1.40; 95%CI = 1.01–1.79; *P* = 0.007), renal morbidity (AOR = 1.23; 95%CI = 1.03–1.45; *P* = 0.019) and serious infection (AOR = 1.31; 95%CI = 1.07–1.60; *P* = 0.009).

**Conclusion:**

Perioperative RBC transfusion in isolated CABG patients is associated with increased risks of developing adverse events such as prolonged ventilatory support, cardiac morbidity, renal morbidity and serious infection.

## Introduction

Coronary artery disease (CAD) is the leading cause of mortality worldwide and in Malaysia (1). According to the World Health Organization, in Malaysia, CAD accounted for 29,400 deaths in 2012, which is equal to 98.9 deaths per 100,000 in the population. CAD also accounts for 20.1% of all mortalities in the country (2). CAD is one of the major burdens of hospitals according to the Ministry of Health. According to the hospital admission records and death certifications, CAD accounted for 6.99% of total hospital admissions and 23.34% of all hospital deaths in 2014 (1).

Treatments for CAD include pharmacological therapy and interventions such as percutaneous coronary intervention (PCI) or coronary artery bypass grafting (CABG). CABG has been the standard of care for the revascularisation of patients with complex CAD since its introduction in 1968 (3). Current evidence has demonstrated a survival benefit with CABG over PCI in patients with three or more vessel disease as well as those with complex coronary artery anatomy. Mohr et al. (4) randomised 1800 patients with left main coronary disease or three-vessel disease to either CABG or PCI. The 5-year study demonstrated a higher survival rate in the CABG group than in the PCI group for patients with complex multi-vessel coronary artery disease.

According to the 2014 European Society of Cardiology (ESC)/European Association for Cardio-Thoracic Surgery (EACTS) guidelines on myocardial revascularisation, the mortality rate associated with CABG is 1%–2%, and the morbidity rate is 1%–2% for each of the following events: stroke; renal, pulmonary and cardiac failure; bleeding; and wound infections (5). The 2011 American College of Cardiology Foundation (ACCF)/American Heart Association (AHA) guidelines for CABG surgery reported that elderly and female patients with diabetes mellitus, chronic obstructive pulmonary disease/ respiratory insufficiency, end-stage renal disease on dialysis, concomitant peripheral vascular disease, previous stroke and reoperative CABG are associated with higher rates of morbidity and mortality (6).

The 2011 ACCF/AHA guidelines for CABG surgery recommended a blood conservation strategy in CABG surgery (6). Blood conservation practices in cardiac surgery were introduced in the 1970s because of the scarcity and cost of this limited resource, the awareness of transfusion-borne infections such as hepatitis B and C and human immunodeficiency virus and the increasing awareness of the immunologic implications of this allogeneic exposure (7).

The rationale for perioperative red blood cell (RBC) transfusions is based on observations that anaemia is an independent risk factor for morbidity and mortality after cardiac operations (8, 9). However, numerous studies have demonstrated that perioperative RBC transfusions in patients undergoing cardiac operations including CABG have been associated with higher rates of morbidity and mortality (10–16). Perioperative blood transfusions have been linked to higher rates of post-operative renal dysfunction (17); neurologic, respiratory and cardiac complications (12–14, 18); serious infection (7, 19, 20); prolonged ventilatory support (12–14); prolonged length of stay (21–23); and short-term and long-term survival (24–31).

The aims of this study are to determine the association between perioperative packed RBC transfusions and adverse outcomes after isolated CABG and to further determine any incremental risk associated with each unit of packed RBC transfusions on morbidity after CABG.

## Methodology

### Study Design

This was a retrospective medical record review and a single-centre study at Hospital Universiti Sains Malaysia (HUSM), Kelantan, Malaysia. The study subjects were patients who were diagnosed with CAD and who underwent isolated CABG at HUSM from 1 January 2013 until 31 December 2017. All procedures were performed by the same two cardiothoracic surgeons. The study protocol was reviewed and approved by the Human Research Ethics Committee of Universiti Sains Malaysia (JEPeM). A stratified sampling method was used to select samples.

Patients who fulfilled the inclusion and exclusion criteria were recruited in our study.

Inclusion criteria:

1. Patients who underwent isolated CABG at HUSM from 1 January 2013 until 31 December 2017
2. Patients more than 18 years old

Exclusion criteria:

1. Patients who underwent combination surgery (e.g. CABG and valve replacement)
2. Patients who underwent isolated CABG with missing or incomplete data

Patients’ case notes were reviewed, and the information collected included patient demographics, comorbidities, cardiac status [left ventricular ejection fraction (LVEF) and New York Heart Association (NYHA) classification], clinical presentation, diseased coronary vessel/ vessels, laboratory parameters (haemoglobin, haematocrit, blood urea and creatinine level), American Society of Anaesthesiologists (ASA) classification, operative information (duration of surgery, operative status, number of bypasses performed, cardiopulmonary bypass time, aortic cross clamp time, and intra-operative and immediate post-operative complications), number of packed RBC transfusions, complications post-surgery (prolonged ventilatory support, cardiac morbidity, neurologic morbidity, renal morbidity, serious infection and mortality) and re-operation. Based on the EuroSCORE measurement, LVEF was classified as normal (LVEF > 50%), moderate (LVEF 31%–49%) or poor (LVEF < 30%) (32).

Data were grouped according to patients who received perioperative RBC transfusions and those who did not. The outcome of interest was complications (prolonged post-operative ventilatory support, cardiac morbidity, neurologic morbidity, renal morbidity, serious infection and post-operative mortality) after CABG among the two groups of subjects.

### Operational Definitions

The terms used in this study were defined as follows:

1. Perioperative RBC transfusion, defined as a transfusion of RBCs during the preoperative, intra-operative and/or postoperative period. Autologous blood transfusions were excluded.
2. Prolonged post-operative ventilatory support, defined as mechanical ventilatory support for more than 72 h post-operatively.
3. Cardiac morbidity, defined as a low cardiac index (1.8 L/min/m2) despite adequate fluid replacement and administration of high-dose inotropic agents for > 4 h or post-operative myocardial infarction.
4. Neurologic morbidity, defined as focal or global neurologic deficits (stroke), as evidenced by clinical or imaging (brain CT) findings.
5. Renal morbidity, defined as new onset of renal failure requiring dialysis or acute kidney injury defined by the Kidney Disease Improving Global Outcomes (KDIGO) criteria:
  - Increase in serum creatinine by 0.3 mg/ dL or more within 48 hours or
  - Increase in serum creatinine to 1.5 times the baseline or more within the last 7 days or
  - Urine output less than 0.5 mL/kg/h for 6 h

1. Serious infection, defined as sepsis syndrome, septic shock, pneumonia, mediastinitis, or sternal or leg wound infection. In addition, the diagnosis of sepsis included organisms isolated from the cultures along with elevated temperature and white blood cell counts.
2. Mortality, defined as in-hospital or 30-day mortality.

### Statistical Analysis

Data were analysed using Statistical Package for the Social Sciences (SPSS) version 24. Data were double checked and cleaned to screen for errors or missing values. Baseline demographics and information of the patients were described, categorical variables were presented as frequencies (percentages), and continuous variables were presented as the mean ± standard deviation (SD).

Data were divided into two groups (patients who received perioperative RBC transfusions and patients who did not); Pearson’s chi-square test or Fisher’s Exact test for categorical variables and independent-samples *t*-test for continuous variables were used to assess the differences in the characteristics between these two groups. Pearson’s chi-square test or Fisher’s Exact test were used to determine the association between perioperative RBC transfusions and each adverse outcome post-CABG. Pearson’s chi-square test was used if the expected count of < 5 was < 20%, and Fisher’s Exact test was used if the expected count of < 5 was > 20%. Adverse outcomes that were significantly associated with perioperative RBC transfusions were further studied for the risks associated with each unit of packed RBC transfusions. Binary logistic regression was used to determine the adjusted odds ratio (AOR) of perioperative RBC transfusion in association with the studied adverse outcomes. Simple logistic regression was used in univariable analysis to determine variables that were significantly associated with the adverse outcome, and these variables were entered into multiple logistic regression. The forward and backward stepwise likelihood ratio (LR) method was used to determine the AOR of factor/factors that significantly increased the risk of the adverse outcome. Multi-collinearity and two-way interactions between variables were checked. Hosmer and Lemeshow test, the area under the receiver operating characteristics (ROC) curve and classification tables were used to test for goodness of fit. AORs with 95% confidence intervals (CIs) and corresponding *P*-values were obtained. A *P*-value of < 0.05 was accepted to be statistically significant.

## Results

**Figure 1:**
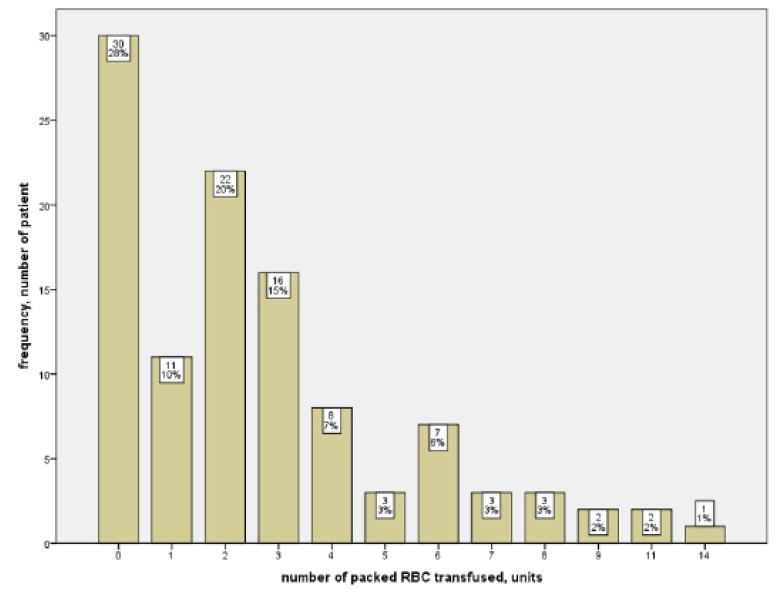
Number of packed red bloods cells tranfused in units for patients who underwent isolated coronary artery bypass grafting

A total of 108 patients who had isolated CABG performed at HUSM, Kelantan, Malaysia, from 1 January 2013 until 31 December 2017 were included in this study. According to the descriptive analysis, 83 (76.9%) patients were male and 25 (23.1%) were female. For the ethnicity, 94 (87%) patients were Malaysian, 12 (11.1%) patients were Chinese, and 2 (1.9%) patients were Indian. A total of 68 (63%) patients were smokers/ex-smokers, and 78 (72.2%) patients received perioperative RBC transfusions, with 2 units being the most common (Figure 1). The majority of the procedures, 105 (97.2%), were elective, and only 3 (2.8%) were emergency procedures. Intra-operative and immediate post-operative complications included bleeding in 1 (0.9%) patient, cardiac arrhythmia in 1 (0.9%) patient and poor cardiac contractility in 2 (1.9%) patients. There were 2 (1.9%) patients who required re-operation. Post-operative complications included prolonged ventilatory support in 17 (15.7%) patients, cardiac morbidity in 11 (10.2%) patients, neurologic morbidity in 2 (1.9%) patients, renal morbidity in 23 (21.3%) patients, serious infection in 17 (15.7%) patients and mortality in 5 (4.6%) patients.

Comparing the transfused and non-transfused groups (Table 1), patients who received perioperative RBC transfusions were older (transfused versus non-transfused: 60 years versus 57 years, *P* = 0.014). Female patients were more likely to receive transfusions than male patients (23.1% versus 0%, *P* < 0.001). The majority of the patients were Malaysian (87.2% versus 86.7%, *P* = 0.764), with no difference between the groups. Lower body mass index (BMI) was observed in the transfused group than in the non-transfused group (26.1 versus 28.8, *P* = 0.005). Patients with a comorbidity of diabetes mellitus (61.5% versus 40%, *P* = 0.044), hypertension (93.6% versus 76.7%, *P* = 0.019), and chronic kidney disease (CKD)/ end-stage renal disease (ESRD) (29.5% versus 10%, *P* = 0.034) and patients with higher NYHA class (III or IV) (26.9% versus 3.3%, *P* = 0.006) and higher ASA class (III and above) (87.2% versus 66.7%, *P* = 0.014) were more likely to have perioperative RBC transfusions. Preoperatively, patients who received transfusions had significantly lower haemoglobin levels (12.7 versus 15.2, *P* < 0.001), lower haematocrit levels (38.1 versus 44.3, *P* < 0.001) and higher blood urea levels (7.3 versus 5.5, *P* = 0.034) compared to patients who did not receive transfusions. No significant differences in the duration of surgery, cardiopulmonary bypass time, aortic cross clamp time, number of grafts used, intra-operative/ immediate post-operative complication rate or re-operation rate were observed between the groups.

According to the univariate analysis (Table 2), patients who received perioperative RBC transfusions were significantly more likely to have prolonged ventilatory support (21.8% versus 0%, *P* = 0.003), cardiac morbidity (14.1% versus 0%, *P* = 0.032), renal morbidity (28.2% versus 3.3%, *P* = 0.005) and serious infection (20.5% versus 3.3%, *P* = 0.037) than patients who did not receive transfusions. Neurologic morbidity (2.6% versus 0%, *P* = 1.000) and mortality rate (6.4% versus 0%, *P* = 0.037) were also higher in the transfused group than in the non-transfused group, but the differences were not statistically significant. Using logistic regression, the risk-adjusted probability of developing each adverse outcome as a function of RBCs was modelled (Tables 3–6). When adjusted for other factors, we found that, with an increase in one unit of packed RBC transfusions, patients had 1.45 times the odds of developing prolonged ventilatory support (95%CI, 1.20–1.77, *P* < 0.001); similar results were found with the other outcomes, with 1.40 times the odds of cardiac morbidity (95%CI, 1.01–1.79; *P* = 0.007), 1.23 times the odds of renal morbidity (95%CI, 1.03–1.45; *P* = 0.019), and 1.31 times the odds of serious infection (95% CI, 1.07–1.60; *P* = 0.009).

## Discussion

The high prevalence of CAD worldwide and in Malaysia (2), with the increasing incidence of CAD over the years, has contributed to the major healthcare burden in the country (1). We sought to study the risk factors associated with morbidity and mortality of CABG because CABG is the main surgical intervention for CAD. Several studies have demonstrated the adverse outcomes associated with RBC transfusion in cardiac surgery and critically ill patients, but there is a lack of studies conducted in our country and in this region that have evaluated the adverse outcomes associated with transfusion of RBCs in CABG. Thus, this study was designed to compare the outcomes between patients who received perioperative RBC transfusions and those who did not. The results of this study showed that perioperative RBC transfusion is associated with adverse outcomes such as prolonged ventilatory support, cardiac morbidity, renal morbidity and serious infection. There is also a dose-dependent, increased risk for these complications with each unit of packed RBC transfusions.

According to the results of this study, among patients who underwent isolated CABG, the percentage of patients who required prolonged ventilatory support was 15.7%; the percentage of those who developed cardiac morbidity, such as those with a low cardiac index or myocardial infarction, was 10.2%; the percentage of those with neurologic morbidity, such as stroke, was 1.9%; the percentage of those with renal morbidity (new onset renal failure requiring dialysis or acute kidney injury) was 21.3%; the percentage of those with serious infection, including lung infection, mediastinitis, or sternal or leg wound infection was 15.7%; and the percentage of those with 30-day/in-hospital mortality was 4.6%. This study reported a higher incidence of these complications in our centre than other studies in other centres. According to the 2014 ESC/EACTS guidelines on myocardial revascularisation, the early clinical outcome 3 months after CABG is characterised by a 1%–2% mortality rate and a 1%–2% morbidity rate for each of the following events: stroke; renal, pulmonary and cardiac failure; bleeding; and wound infection (5).

RBC transfusion in cardiac surgery, including CABG, is a common practice as seen in other surgical disciplines; the most common indications for perioperative RBC transfusion are pre-operative symptomatic anaemia, intra-operative excessive/life threatening bleeding or post-operative low haemoglobin levels. In our centre, the decision for blood transfusion was based on the patient’s clinical status (symptomatic anaemia, intra-operative or post-operative excessive/life threatening bleeding, coagulopathies) and/or haemoglobin < 8 g/dL. However, throughout the years, a growing amount of literature has shown that RBC transfusion is associated with morbidity and short-term and long-term mortality after CABG (33–35). Although RBC transfusions may certainly have life-preserving value, the impact of smaller quantities of RBCs in a non-emergent setting (i.e., asymptomatic anaemia/bleeding) has not been well documented. These small-volume transfusions are more discretionary and therefore potentially avoidable. A common rationale for RBC transfusion is to increase oxygen delivery to organ tissues sensitive to ischaemia in patients with haematocrit or haemoglobin levels below a predetermined and usually arbitrarily set lower limit. However, well-described changes in RBC morphology and the significant depletion of 2,3-diphosphoglycerate and nitric oxide levels that occur during storage are known to profoundly limit the capacity of these RBCs to carry and deliver oxygen to tissues. The accumulation of immunomodulating bioactive substances released from leukocytes to the storage medium and the transfusion of white blood cell-containing allogeneic RBC products have been associated with an increased risk of post-operative infection in cardiac surgery (15, 20). Thus, all of these outcomes have called into question the benefit of many of these types of transfusions.

This study demonstrated that perioperative RBC transfusion is significantly associated with prolonged ventilatory support (*P* = 0.003), renal morbidity (*P* = 0.005), cardiac morbidity (*P* = 0.032) and serious infection (*P* = 0.037) after isolated CABG. This result corresponds to the results of a few published studies. Koch et al. in their observational cohort study in 2006 reported that perioperative RBC transfusion was associated with an increased risk of post-operative prolonged ventilatory support after isolated CABG (13). In 2012, Karkouti analysed 22 observational studies and concluded that perioperative blood transfusion was an independent risk factor for acute kidney injury (renal morbidity) in cardiac surgery (17). Surgenor et al. reported that intra-operative RBC transfusion during coronary artery bypass graft surgery increased the risk of post-operative low-output heart failure (cardiac morbidity) from their prospective observational study in 2006 (18). In a cohort study conducted in 389 patients by Rogers et al., their results showed that the transfusion of allogeneic blood products in cardiac surgery increased the risk of infection (19). Although the percentages of neurologic morbidity (transfused versus non-transfused, 2.6% versus 0%, *P* > 0.05) and post-operative mortality (6.4% versus 0%, *P* > 0.05) were higher in the transfused group than in the non-transfused group, the differences were not statistically significant. This is in contrast with results shown by Koch et al. and Murphy et al., who revealed that RBC transfusion in patients with cardiac surgery/isolated CABG is associated with neurologic adverse events and postoperative mortality (13, 14).

The number/unit of transfused RBCs is an independent risk factor for worse outcomes, including mortality. In a retrospective cohort study of 11,963 patients who underwent isolated CABG surgery, Koch et al. showed that perioperative RBC transfusion was associated with a dose-dependent increased risk of postoperative cardiac complications, serious infection, renal failure, neurologic complications, overall morbidity, prolonged ventilatory support, and in-hospital mortality (13). In a similar study, Murphy et al. showed that RBC transfusion was strongly associated with infection and postoperative ischaemic morbidity, hospital stay, increased early and late mortality, and hospital costs, and a strong dose-response relationship was present (14). Our study also showed a dose-dependent increased risk of post-operative prolonged ventilatory support, cardiac events, renal complications and serious infection in isolated CABG patients with perioperative RBC transfusions. With a one-unit increase in packed RBC transfusions, there was an increased risk for prolonged ventilatory support (AOR, 1.45, 95%CI, 1.20–1.77, *P* < 0.001), cardiac morbidity (AOR, 1.40, 95%CI, 1.01–1.79; *P* = 0.007), renal morbidity (AOR, 1.23, 95%CI, 1.03–1.45; *P* = 0.019) and serious infection (AOR, 1.31, 95%CI, 1.07–1.60; *P* = 0.009).

In addition, we found that perioperative RBC transfusions were more common with characteristics of older age, female sex and low BMI as well as in patients with diabetes mellitus, hypertension, CKD/ESRD, a higher NYHA class (III or IV), low haemoglobin and haematocrit levels, a high blood urea level and a higher ASA class (III and above). However, these results were only found with univariable analyses. According to the literature, predictors for perioperative transfusion are re-operation, age, female sex, pre-operative haematocrit level, low ejection fraction, pre-operative serum creatinine level (≥ 1.3 mg/dL), and low body weight (13, 36–39).

Increasing evidence has shown that blood-conserving strategies are beneficial in cardiac surgery (40–46). As evidenced by a study conducted by Hajjar et al. in 2010 with a the transfusion requirement after cardiac surgery (TRACS) randomised controlled trial (RCT), 502 patients who underwent cardiac surgery were randomly assigned to a liberal strategy of blood transfusion (to maintain a haematocrit level ≥ 30%) or to a restrictive strategy (haematocrit level ≥ 24%); the study results showed that among patients undergoing cardiac surgery, the use of a restrictive perioperative transfusion strategy compared with a more liberal strategy resulted in non-inferior rates of the combined outcome of 30-day all-cause mortality and severe morbidity (47).

This study has its limitations because it was a retrospective, non-randomised study, and it introduced a substantial possibility of selection bias. It was a single-centre study that only involved the population of a single state (Kelantan) of Malaysia; the results could have been more representative if the study was performed at multiple centres across the nation. As is typical with a retrospective review of records, we were unable to obtain certain useful information, for example, the EuroSCORE risk stratification model, to assess the operative risk; this limitation could have been alleviated if the study was designed prospectively.

## Conclusion

Perioperative RBC transfusion in isolated CABG is associated with adverse outcomes such as prolonged ventilatory support, cardiac morbidity, renal morbidity and serious infection. There is also a dose-dependent increase in the occurrence of these post-operative adverse events associated with packed RBC transfusions. Therefore, a more conservative approach of blood transfusion in cardiac surgery should be taken into consideration.

## Figures and Tables

**Table 1:** Characteristics of patients underwent isolated CABG who received perioperative RBC transfusions and patients who did not

Variables	Transfused (*n* = 78)	Not Transfused (*n* = 30)	*P*-value
**Age**	60 ± 6.9	57 ± 7.3	0.014^a^
> 65	19 (24.4%)	3 (10%)	0.097^b^
**Gender**			
Male	53 (67.9%)	30 (100%)	< 0.001^b^
Female	25 (23.1%)	0 (0%)	
**Race**			
Malay	68 (87.2%)	26 (86.7%)	0.764^b^
Chinese	9 (11.5%)	3 (10%)	
Indian	1 (1.3%)	1 (3.3%)	
**Characters**			
BMI, kg/m^2^	26.1 ± 4.3	28.8 ± 4.3	0.005^a^
Smoker/ex-smoker	42 (53.8%)	26 (86.7%)	0.002^b^
**Co-morbidities**			
Diabetes mellitus	48 (61.5%)	12 (40%)	0.044^b^
Hypertension	73 (93.6%)	23 (76.7%)	0.019^c^
Hyperlipidemia	62 (79.5%)	27 (90%)	0.199^b^
CKD/ESRD	23 (29.5%)	3 (10%)	0.034^b^
Previous stroke	9 (11.5%)	0 (0%)	0.060^c^
Cardiac arrhythmia	3 (3.8%)	0 (0%)	0.559^c^
COAD/asthma	3 (3.8%)	0 (0%)	0.559^c^
**Cardiac Status**			
LVEF > 50%	46 (59%)	23 (76.7%)	0.086^b^
LVEF ≤ 50%	32 (41%)	7 (23.3%)	
NYHA class I or II	57 (73.1%)	29 (96.7%)	0.006^b^
NYHA class III or IV	21 (26.9%)	1 (3.3%)	
**Clinical Presentation**			
Stable angina	5 (6.4%)	3 (10%)	0.683^c^
Unstable angina	34 (43.6%)	13 (43.3%)	0.981^b^
Myocardial infarction	27 (34.6%)	14 (46.7%)	0.248^b^
Congestive cardiac failure	8 (10.3%)	0 (0%)	0.103^c^
Cardiogenic shock	4 (5.1%)	0 (0%)	0.574^c^
**Coronary Artery Stenosis (> 50%)**			
Left main stem	42 (53.8%)	14 (46.7%)	0.504^b^
Left anterior descending artery	74 (94.9%)	30 (100%)	0.574^c^
Left circumflex artery	65 (83.3%)	30 (100%)	0.018^c^
Right coronary artery	69 (88.5%)	29 (96.7%)	0.278^c^
**Laboratory Parameters**			
Haemoglobin, g/dL	12.7 ± 1.9	15.2 ± 1.2	< 0.001^a^
Haematocrit, %	38.1 ± 6.2	44.3 ± 4.6	< 0.001^a^
Blood urea, mg/dL	7.3 ± 4.4	5.5 ± 1.6	0.034^a^
Creatinine, mg/dL	152 ± 117.2	119 ± 22.1	0.132^a^
**ASA Class**			
I or II	10 (12.8%)	10 (33.3%)	0.014^b^
III and above	68 (87.2%)	20 (66.7%)	
**Operative Status**			
Elective	75 (96.2%)	30 (100%)	0.559^c^
Emergency	3 (3.8%)	0 (0%)	
**Surgery**			
Duration, minutes	261 ± 44.4	264 ± 35.9	0.780^a^
Cardiopulmonary bypass time, minutes	114 ± 26.4	105 ± 20.2	0.102^a^
Aortic cross clamp time, minutes	82 ± 19.6	78 ± 16.9	0.332^a^
	3 ± 0.4	3 ± 0.3	0.055^a^
Number of grafts	54 (69.2%)	26 (86.7%)	0.064^b^
LIMA graft used	3 (3.8%)	1 (3.3%)	> 0.95^c^
Intra-operative or immediate			
Post-operative complications	2 (2.6%)	0 (0%)	> 0.95^c^
**Re-operation**			

a Independent-samples t-test applied;

b Pearson chi-square test applied;

c Fisher’s Exact test applied;

BMI = body mass index; CKD = chronic kidney disease; ESRD = end stage renal disease; COAD = chronic obstructive airway disease; LVEF = left ventricular ejection fraction; NYHA = New York Heart Association; ASA = American Society of Anaesthesiologists; LIMA = left internal mammary artery

**Table 2:** Comparison of outcomes between patients who received perioperative RBC transfusions and patients who did not

Variables^a^	Transfused (*n* = 78)	Not transfused (*n* = 30)	Total (*n* = 108)	*P*-value
**Prolonged ventilatory support**				
yes	17 (21.8%)	0 (0%)	17 (15.7%)	0.003^b^
no	61 (78.2%)	30 (25.3%)	91 (84.3%)	
**Cardiac morbidity**				
yes	11 (14.1%)	0 (0%)	11 (10.2%)	0.032^b^
no	67 (85.9%)	30 (100%)	97 (89.8%)	
**Neurologic morbidity**				
yes	2 (2.6%)	0 (0%)	2 (1.9%)	> 0.95^b^
no	76 (97.4%)	30 (100%)	106 (98.1%)	
**Renal morbidity**				
yes	22 (28.2%)	1 (3.3%)	23 (21.3%)	0.005^c^
no	56 (71.8%)	29 (96.7%)	85 (78.7%)	
**Serious infection**				
yes	16 (20.5%)	1 (3.3%)	17 (15.7%)	0.037^b^
no	62 (79.5%)	29 (96.7%)	91 (84.3%)	
**Mortality**				
yes	5 (6.4%)	0 (0%)	5 (4.6%)	0.319^b^
no	73 (93.6%)	30 (100%)	103 (95.4%)	

**Table 3:** Simple and multiple logistic regression analysis of factors associated with prolonged ventilatory support

Odds ratio (OR) of factors^a^ associated with prolonged ventilatory support					
Variable	B^b^	Wald	OR	95%CI^c^	*P*-value
PRBC^d^, units	0.643	5.364	1.90	1.10–3.28	0.021
Blood urea, mg/dL	0.140	5.264	1.15	1.02–1.30	0.022

**Adjusted odds ratio (AOR) of factor associated with prolonged ventilatory support**					

Variable	B	Wald	AOR	95%CI	*P*-value
Constant	−3.006	35.168	0.49		< 0.001
PRBC, units	0.373	14.118	1.45	1.20–1.77	< 0.001
Blood urea, mg/dL	0.299	2.233	1.35	0.91–1.99	0.135

* Forward and backward stepwise likelihood ratio (LR) methods was applied

* No multi-collinearity and no interaction were found

* Hosmer Lemeshow test [Pearson chi-square test (5) = 5.932, *P* = 0.313]

* Classification table 85.2% correctly classified

* Area under receiver operating characteristics (ROC) curve was 0.797, P < 0.001

a All variables in Table 1 were considered in variable selection process

b Regression coefficient;

c Confidence interval;

d Packed red blood cell

**Table 4:** Simple and multiple logistic regression analysis of factors associated with cardiac morbidity

OR of factors^a^ associated with cardiac morbidity					
Variable	B^b^	Wald	OR	95%CI^c^	*P*-value
PRBC^d^, units	0.434	13.782	1.54	1.23–1.94	< 0.001
CKD/ESRD^e^	0.130	5.451	4.62	1.28–16.7	0.020
LVEF^f^ ≤ 50%	1.736	5.962	5.68	1.41–22.88	0.015
NYHA^g^ class 3 or 4	2.761	14.066	15.81	3.74–66.91	< 0.001
CCF^h^	1.932	5.575	6.9	1.39–34.29	0.018
Cardiogenic shock	2.357	4.952	10.56	1.32–84.13	0.026
Blood urea, mg/dL	0.126	4.255	1.13	1.01–1.28	0.039

**AOR of factor associated with cardiac morbidity**					

Variable	B	Wald	AOR	95%CI	*P*-value
Constant	4.360	29.572	0.01		< 0.001
PRBC, units	0.337	7.156	1.40	–1.79	0.007
NYHA class III or IV	2.016	6.251	7.51	1.55–36.44	0.012

* Forward and backward stepwise likelihood ratio (LR) methods was applied

* No multicollinearity and no interaction were found

* Hosmer Lemeshow test [Pearson chi-square test (5) = 4.628, *P* = 0.592]

* Classification table 90.7% correctly classified

* Area under receiver operating characteristics (ROC) curve was 0.915, *P* < 0.001

a All variables in Table 1 were considered in variable selection process

b Regression coefficient;

c Confidence interval;

d Packed red blood cells;

e Chronic kidney disease/end stage renal disease;

f Left ventricular ejection fraction;

g New York Heart Association;

h Congestive cardiac failure

**Table 5:** Simple and multiple logistic regression analysis of factors associated with renal morbidity

OR of factors^a^ associated with renal morbidity					
Variable	B^b^	Wald	OR	95%CI^c^	*P*-value
PRBC^d^, units	0.267	9.987	1.31	1.11–1.54	0.002
Diabetes mellitus	1.629	7.585	5.10	1.60–16.25	0.006
CKD/ESRD^e^	1.974	14.555	7.2	2.61–19.85	< 0.001
Blood urea, mg/dL	0.170	6.607	1.19	1.04–1.35	0.010

**AOR of factor associated with renal morbidity**					

Variable	B	Wald	AOR	95%CI	*P*-value
Constant	−2.527	32.232	0.08		< 0.001
**PRBC, units**	0.203	5.510	1.23	−1.45	0.019
**CKD/ESRD**	1.659	9.339	5.25	1.81–15.22	0.002

* Forward and backward stepwise likelihood ratio (LR) methods was applied

* No multicollinearity and no interaction were found

* Hosmer Lemeshow test [Pearson chi-square test (5) = 4.594, *P* = 0.597]

* Classification table 78.7% correctly classified

* Area under receiver operating characteristics (ROC) curve was 0.792, *P* < 0.001

a All variables in Table 1 were considered in variable selection process

b Regression coefficient;

c Confidence interval;

d Packed red blood cells;

e Chronic kidney disease/end stage renal disease

**Table 6:** Simple and multiple logistic regression analysis of factors associated with serious infection

OR of factors^a^ associated with serious infection					
Variable	B^b^	Wald	OR	95%CI^c^	*P*-value
PRBC^d^, units	0.336	12.503	1.40	1.16–1.69	< 0.001
Diabetes mellitus	1.518	5.138	4.57	1.23–16.97	0.023
LVEF^e^ ≤ 50%	1.117	4.248	3.05	1.06–8.83	0.039
NYHA^f^ class III or IV	1.910	11.190	6.75	2.21–20.66	0.001

**AOR of factor associated with serious infection**					

Variable	B	Wald	AOR	95% CI	*P*-value
Constant	−2.997	34.865	0.05		< 0.001
**PRBC, units**	0.267	6.837	1.31	−1.60	0.009
**NYHA class III or IV**	1.262	3.903	3.53	1.01–12.35	0.048

* Forward and backward stepwise likelihood ratio (LR) methods was applied

* No multicollinearity and no interaction were found

* Hosmer Lemeshow test [Pearson chi-square test (5) = 5.932, *P* = 0.270]

* Classification table 88.9% correctly classified

* Area under receiver operating characteristics (ROC) curve was 0.756, *P* = 0.001

a All variables in Table 1 were considered in variable selection process

b Regression coefficient;

c Confidence interval;

d Packed red blood cells;

e Left ventricular ejection fraction;

f New York Heart Association
